# Development and Characterization of Microsatellite Genetic Markers for *Hyalomma rufipes*, a Tick Vector of Crimean‐Congo Hemorrhagic Fever Virus

**DOI:** 10.1002/ece3.73064

**Published:** 2026-02-05

**Authors:** Hamza Ahmad, Winnifred Aool, Victor Anyango, Teddy M. Nakayaki, Francis Mulwa, Betty Chelangat, Julius J. Lutwama, Jonathan K. Kayondo, Martin Lukindu, James Mutisya, Joel Lutomiah, Lisa E. Hensley, Lee W. Cohnstaedt, Maria G. Onyango, Corey L. Brelsfoard

**Affiliations:** ^1^ Department of Biological Sciences Texas Tech University Lubbock Texas USA; ^2^ Department of Entomology Uganda Virus Research Institute Entebbe Uganda; ^3^ Centre for Virus Research Kenya Medical Research Institute Nairobi Kenya; ^4^ USDA Foreign Arthropod‐Borne Animal Diseases Research Unit Manhattan Kansas USA

**Keywords:** East Africa, genetic markers, population genetics, tick borne disease

## Abstract

*Hyalomma rufipes* is a widely distributed tick species and a competent vector of Crimean‐Congo Hemorrhagic Fever Virus (CCHFV), a serious zoonotic pathogen endemic to over 30 countries. Despite the epidemiological importance of CCHFV and *H. rufipes* in East Africa, little is known about the genetic structure and movement of *H. rufipes* populations, limiting the understanding of CCHFV transmission dynamics in this region. This study developed and characterized 14 polymorphic microsatellite markers to support population genetic studies of *H. rufipes* . *H. rufipes* ticks were collected from livestock in Garissa and Isiolo counties in northern Kenya. Morphological identification was confirmed using 16S rRNA Sanger sequencing and phylogenetic analysis. Low‐pass whole genome sequencing was performed on representative samples, and the Quality and Diversity of DNA (QDD) pipeline was used to identify and design microsatellite primers. Of 59,201 candidate loci, 30 were selected for initial screening; 14 loci consistently amplified and were polymorphic. These included mostly tetranucleotide repeats and showed high allelic richness and gene diversity. Several loci showed signs of null alleles, but no evidence of stuttering or allelic dropout was found. These newly developed microsatellite markers provide a valuable tool for investigating *H. rufipes* population dynamics and dispersal, with the ultimate goal of understanding CCHFV transmission dynamics in East Africa.

## Graphical abstract

This study developed and validated 14 polymorphic microsatellite markers to investigate the population genetics of Hyalomma rufipes, a tick species that transmits Crimean‐Congo Hemorrhagic Fever Virus (CCHFV) in East Africa. Ticks collected from livestock in northern Kenya were morphologically and genetically identified, followed by genome sequencing and microsatellite marker design. The markers demonstrated high genetic diversity and will aid in understanding the movement and population structure of H. rufipes, which is crucial for mapping CCHFV transmission in the region.

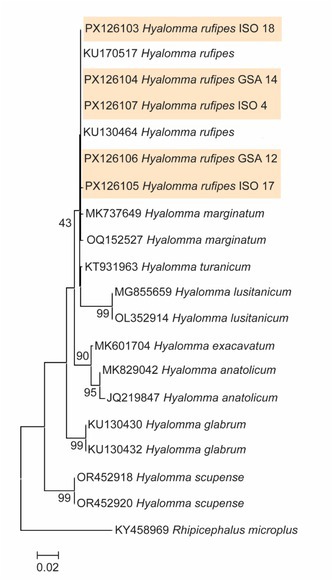

## 1 Introduction

Ticks are an important vector of viral, bacterial, and protozoan pathogens that affect both human and animal health (Chiuya et al. 2021; de la Fuente et al. 2008). Crimean‐Congo Hemorrhagic Fever Virus (CCHFV) is a prevalent tick‐borne virus that affects humans and is endemic in over 30 countries (Al‐Abri et al. 2017). The hard tick *Hyalomma rufipes* is a confirmed vector of CCHFV (Al‐Abri et al. 2017; Nasirian 2022; Sang et al. 2011; Addae 2018; Nabeth et al. 2004; Zeller et al. 1997; Okely et al. 2020; Mancuso et al. 2019). Other pathogens *H. rufipes* is known to transmit include *Rickettsia aeschlimannii* and *Babesia occultans* (Bonnet et al. 2023). *H. rufipes* is a two‐host tick that commonly feeds on cattle, sheep, goats, camels, and horses, while immatures feed on hares and birds (Chen et al. 2012). *H. rufipes* is found across Africa, the Middle East, and central Asia, but its range has expanded into Europe by traveling on migratory birds (Chen et al. 2012; Onyiche and MacLeod 2023; Mwangi et al. 1985; Teel et al. 1988; Bryson et al. 2002; Hove et al. 2008; Tomassone et al. 2012; Hassan et al. 2013; Omondi et al. 2017; Chitimia‐Dobler et al. 2019; Grandi et al. 2020; Shuaib et al. 2020; Springer et al. 2020).

Travel and animal trade can affect pathogen spread by moving infected animals and animals with tick infestations harboring pathogens such as CCHFV. This is concerning in endemic areas such as in East Africa, as livestock have been found to be heavily infested with ticks in peri‐urban areas and are often translocated in the Ugandan‐Kenya cattle corridor. (Chiuya et al. 2021; Kilpatrick and Randolph 2012; Mossel et al. 2017; Nyaruaba et al. 2019; Fèvre et al. 2005, 2006; Sang et al. 2006). CCHFV infected ticks from the genus *Hyalomma* collected in Kenya and outbreaks of CCHFV have previously been reported (Chiuya et al. 2021; Dunster et al. 2002). However, little is known about how ticks such as *H. rufipes* disperse across landscapes or how populations are structured in CCHFV endemic areas of East Africa. Developing microsatellite markers for *H. rufipes* in this region would provide an important tool for tracking tick movement and population connectivity, and aid in the understanding of CCHFV transmission dynamics.

Microsatellite markers are a genetic tool that has been used to elucidate reproductive strategies, dispersal mechanisms, population size and structure of organisms and are widely accepted for use in population genetic studies (Araya‐Anchetta et al. 2015; Barbará et al. 2007; Väli et al. 2008). Microsatellite regions are repeats of two to five nucleotides and are codominant and generally highly polymorphic in natural populations (Araya‐Anchetta et al. 2015). Microsatellite markers have been successfully characterized for a number of insects and arthropods, including ticks from multiple genera including: *Dermacentor, Ixodes, Rhipicephalus* and *Hyalomma* (Araya‐Anchetta et al. 2015; Leo et al. 2012; Dharmarajan et al. 2009a, 2009b; Van Houtte et al. 2013; Delaye et al. 1998; Røed et al. 2006; Fagerberg et al. 2001; Mccoy and Tirard 2000; Araya‐Anchetta 2012; Chigagure et al. 2000; Cutullé et al. 2009; Koffi et al. 2006; Busch et al. 2014; Hekimoglu et al. 2019). While five microsatellite markers are available for closely related species such as *Hyalomma marginatum*, no microsatellite markers have been characterized for *H. rufipes* (Hekimoglu et al. 2019, 2020). Here we utilized high throughput genome sequencing and the QDD pipeline to locate and characterize microsatellite markers for *H. rufipes* . The development of microsatellite markers for *H. rufipes* will ultimately help to inform surveillance strategies, identify high‐risk transmission corridors, and support targeted disease and tick control efforts in endemic areas such as East Africa.

## 2 Materials and Methods

### 2.1 Tick Sampling

Tick samples (2335) were collected during the short rainy season between (November and December 2023). Samples were collected from Garissa (E 39.1920841, N 0.7570419) and two sites in Isiolo (E 39.062222, N 0.926111) (E 39.129444, N 0.86778) in Northern Kenya. Adult tick samples were passively collected from different predilection sites of livestock including cattle, sheep, camels, and goats using sterile forceps. The collected ticks were immersed in a labeled tube with DNA/RNA Shield reagent (Zymo research, CA, USA) and stored in liquid nitrogen for transportation. All collected ticks were transported to the Kenya Medical Research Institute (KEMRI) for morphological identification and further processing. Ticks were sorted on a chilled table under a stereo microscope (Leica M80) and morphologically identified to species level using taxonomic keys (Walker 1974). Thirty‐six total samples from Garissa and Isiolo (eighteen from each region) were identified as *H. rufipes* and were used to develop and characterize the identified microsatellite regions. The Isiolo collection consisted of four males and fourteen females, and the Garissa collection consisted of nine males and females.

### 2.2 DNA Isolation

For low‐pass whole genome sequencing, DNA was extracted from a pooled sample of the legs of four *H. rufipes* females using a DNeasy Blood and Tissue kit (QIAGEN, Hilden, Germany) following the manufacturer's instructions with a few modifications. Legs of four adult female ticks were utilized to avoid host contamination since ticks were collected off of their vertebrate host and likely contained host blood. In brief, the modifications to the DNeasy isolation protocol included a 2 min homogenization step with a 2 mm Zirconia bead and a tissue homogenizer (Biospec products), followed by an extension of the tissue lysis incubation period to > 8 h, and ending with a final elution step consisting of 100 μL of AE buffer. Using the same aforementioned protocol, DNA was also isolated from whole ticks for 16 s sequencing to confirm tick species identification genotyping using the identified microsatellite regions.

### 2.3 *H. rufipes* Morphological Identification Confirmation Using 16 s Sanger Sequencing

Morphological identification was confirmed using PCR and Sanger sequencing using previously published primers for the 16S rRNA gene [16S + 1 (5′‐CTG CTC AAT GAT TTT TTA AAT TGC TGT GG‐3′) and 16S−1 (5′‐CCG GTC TGA ACT CAG ATC AAG T‐3′)] (Black and Piesman 1994). PCR conditions consisted of a total reaction volume of 25 μL: 12.5 μL of template mastermix (Taq plus 2 × 1.5 mM MgCl_2_ VWR LifeScience PA), 0.5 μL of forward primer (10 μM), 0.5 μL of reverse primer (10 μM), 1 μL of template DNA and 10.5 μL of molecular grade water. All PCR reactions used the following thermocycler parameters: denaturation at 95°C for 3 min, 34 cycles of denaturation at 95°C for 30 s, annealing at 55°C for 30 s, and extension at 72°C for 1 min. The final extension was set at 72°C for 5 min before a 12°C indefinite hold step. PCR amplification was confirmed using a 1.5% agarose gel and 1× TBE with 10 μL of 10,000× gel red in water (EMD Millipore, MA, USA). Successful amplifications were cleaned using ExoSAP‐IT (Applied Biosystems, Waltham, MA, USA) and processed for Sanger sequencing by Genewiz (South Plainfield, NJ, USA). A 100% BLAST identification match was used to confirm the species of the tick samples. To examine the evolutionary relationships to other closely related species and to aid in confirming the identity of *H. rufipes* MEGA version 11 was used to build a phylogenetic tree using a 387 bp sequence of 16 s rRNA. The maximum likelihood tree was constructed using the Tamura‐Nei substitution model with 10,000 bootstrap replicates (Tamura et al. 2021; Tamura and Nei 1993).

### 2.4 Low‐Pass Whole Genome Sequencing

Two replicate samples consisting of isolated DNA from the legs of four adult female *H. rufipes* were used for low‐pass genome sequencing. DNA quality was checked using a Qubit prior to library preparation. The DNA was enzymatically fragmented and the library prepared using end‐repair and A‐tailing chemistry. Sequencing was conducted with low‐pass whole genome sequencing at Genewiz using the Illumina Hiseq platform with paired end reads with 2 × 150 bp chemistry. (Azenta Life Sciences, Burlington MA, USA). The sequences were not trimmed since adapter clipping is irrelevant if the sequences are assembled for step one of the QDD pipeline.

### 2.5 Microsatellite Region Detection and Primer Design

PEAR was used to merge the paired‐end reads of the genome into a single input file containing contigs (Zhang et al. 2014). To increase the chances of selecting microsatellite markers with polymorphism, sequences with at least eight repeats were chosen for further analysis (Bagshaw 2017; Ananda et al. 2013). The QDD pipeline was ran on Linux as the command line version and consisted of three steps from microsatellite repeat region identification to oligonucleotide design and selection. Step one of the pipeline involved parsing the genome for microsatellites based on the QDD default parameters. The default values utilized for step one were as follows: flanking region length of 200, minimum sequence length 80, and five as the minimum repeat number for microsatellite detection. Step two eliminated grouped reads, low complexity sequences, and intra‐sequence repetitions to remove transposons and minisatellites (Meglécz et al. 2014). Singleton sequences were kept for primer design (Meglécz et al. 2014). Step three consisted of designing the oligonucleotide sequences for microsatellite regions by selecting the size of PCR product and ensuring the microsatellite target was between primer binding sites (Meglécz et al. 2014). The parameters were set to prioritize pure microsatellites, which lack both nanosatellites (three to four repeats of a two to six bp motif) and homopolymers (a base repeating at least five times). Default primer design parameters for Primer 3 (version 2) were utilized. Additional criteria for primer selection include picking pure microsatellites, design parameter as “A”, and lastly, with as low of a primer 3 penalty score as possible (Meglécz et al. 2014). The criteria for a primer to be designated as “A” is as follows: no homopolymer in the flanking region and primer, no other microsatellite target allowed in the region, no nanosatellites in the primer or flanking region, and the target microsatellite is not allowed to be a compound. Thirty initial loci were identified and the designed primers tested for successful amplification using eighteen individuals from Garissa and 18 individuals from Isiolo (Table S1). Thesex and collection location of each individual tick are listed in File S2.

### 2.6 PCR Conditions and Genotyping

To test primer design and to amplify the selected microsatellite regions, PCR consisted of a total reaction volume of 25 μL: 12.5 μL of template mastermix (Taq plus 2 × 1.5 mM MgCl_2_ VWR LifeScience PA), 0.5 μL of forward primer (10 μM), 0.5 μL of reverse primer (10 μM), 1 μL of template DNA and 10.5 μL of molecular grade water as the reagents per reaction. All reactions were singleplexes. All PCR reactions used the following thermocycler parameters: denaturation at 95°C for 3 min, 34 cycles of denaturation at 95°C for 30 s, annealing at 55°C for 30 s, and extension at 72°C for 1 min. The final extension was set at 72°C for 5 min before a 12°C indefinite hold step. To confirm amplification, amplicons were run on 1.5% agarose with 1× TBE and 10 μL of 10,000× GelRed in water (EMD Millipore, MA, USA). Loci with single, clear bands were kept for further analysis. Exosap‐IT (Thermofisher Scientific, Waltham, MA, USA) was used to purify amplicons for fragment analysis. Fragment analysis was performed by Genewiz (Azenta Life Sciences, South Plainfield, NJ, USA) with an ABI 3730 XL DNA Analyzer (Thermofisher Scientific, Waltham, MA, USA). Forward primers were labeled with 5′ ATTO 565, 5′ ATTO 550, 5′ Yakima Yellow, and 5′ 6‐FAM dyes (Table 1). Peak scanner (Thermofisher Scientific, Waltham, MA, USA) was used to analyze amplicon sizes.

**TABLE 1:** Microsatellite primer sequences, repeat motif, product size range, and annealing temperature for the 14 polymorphic microsatellite loci selected for *H. rufipes* .

Loci name	Forward primer (5′‐3′)	Reverse primer (5′‐3′)	Repeat motif	Product size range (bp)	Annealing temperature, °C
Hrms‐4	^1^ACATCAGCAACACAACGCAC	GGAGCCTACATAATGCGCCT	(AAAC)_13_	168–344	55°C
Hrms‐5	^2^TTACGCTCACAGTGACACCC	ATCGCGTGGCTACCTATGTG	(AGAT)_21_	220–392	55°C
Hrms‐6	^1^AAGCGATGGCAGTGTCGTTA	AACGTGACGCAGCAAGTTTC	(AGAT)_10_	212–360	55°C
Hrms‐7	^3^GGTCGTGTCAGCCAACCATA	GCCGTCAAACAAGGTGTCAC	(AGAT)_13_	160–220	55°C
Hrms‐8	^4^CGCCAACATCAGCAACACAA	AGGAGCCTACATAATGCGCC	(AAAC)_13_	160–280	55°C
Hrms‐9	^2^CAGCCGAGTACGATGTCCTC	TGACACCAGTGGCGGTATTC	(AGAT)_11_	196–352	55°C
Hrms‐10	^3^AACGGTGAGATGCATGGGTT	GCAAGTTTCAACGAACGCCT	(AGAT)_9_	180–328	55°C
Hrms‐11	^1^ATAGCGCACAGTACTCGAGC	AAAGCGGTGGATGCCTGTTA	(AAT)_13_	141–345	55°C
Hrms‐13	^4^TCTAGCAGGGCTCAGGCTAA	ACCATTCGACCCTGCTTGAG	(AAAG)_8_	136–244	55°C
Hrms‐14	^3^ATGGCTGTAGCGATGGTACG	ACAACAGCTCCATTCTCCGG	(AG)_9_	112–150	55°C
Hrms‐16	^2^GCGCCCTTCTCCTAACCTTT	CGAACCCACCTTCTTCGACA	(AAAT)_13_	168–272	55°C
Hrms‐21	^4^AAGCGGAGTTCCCTAACACG	TAAGCTCGAACACGCTGGTT	(AAAG)_8_	140–220	55°C
Hrms‐24	^2^AAGCGGAGTTCCCTAACACG	GCAGTGGTATATCGCTGGCT	(AAAG)_8_	160–256	55°C
Hrms‐28	^4^GGTCGTGTCAGCCAACCATA	GGGTTAACCGGTGTGGCATA	(AGAT)_13_	160–260	55°C

*Note:* Primer sequences of all microsatellite loci. 1 is 5′ ATTO 565, 2 is ATTO 550, 3 is Yakima Yellow, 4 is 5′ 6‐FAM. Forward primers were labeled with these dyes.

### 2.7 Population Genetics Analysis

Exact tests for Hardy‐Weinburg equilibrium, observed heterozygosity, and expected heterozygosity were calculated with GENEPOP (Rousset 2008). Fstat was utilized to compute F‐statistics, allelic richness, and gene diversity with default Markov chain parameters (Goudet 2002). Microchecker was used for null allele detection, allelic dropout, and stuttering (Van Oosterhout et al. 2004).

## 3 Results

### 3.1 *H. rufipes* Morphological Identification Confirmation Using 16 s Sanger Sequencing

**FIGURE 1:**
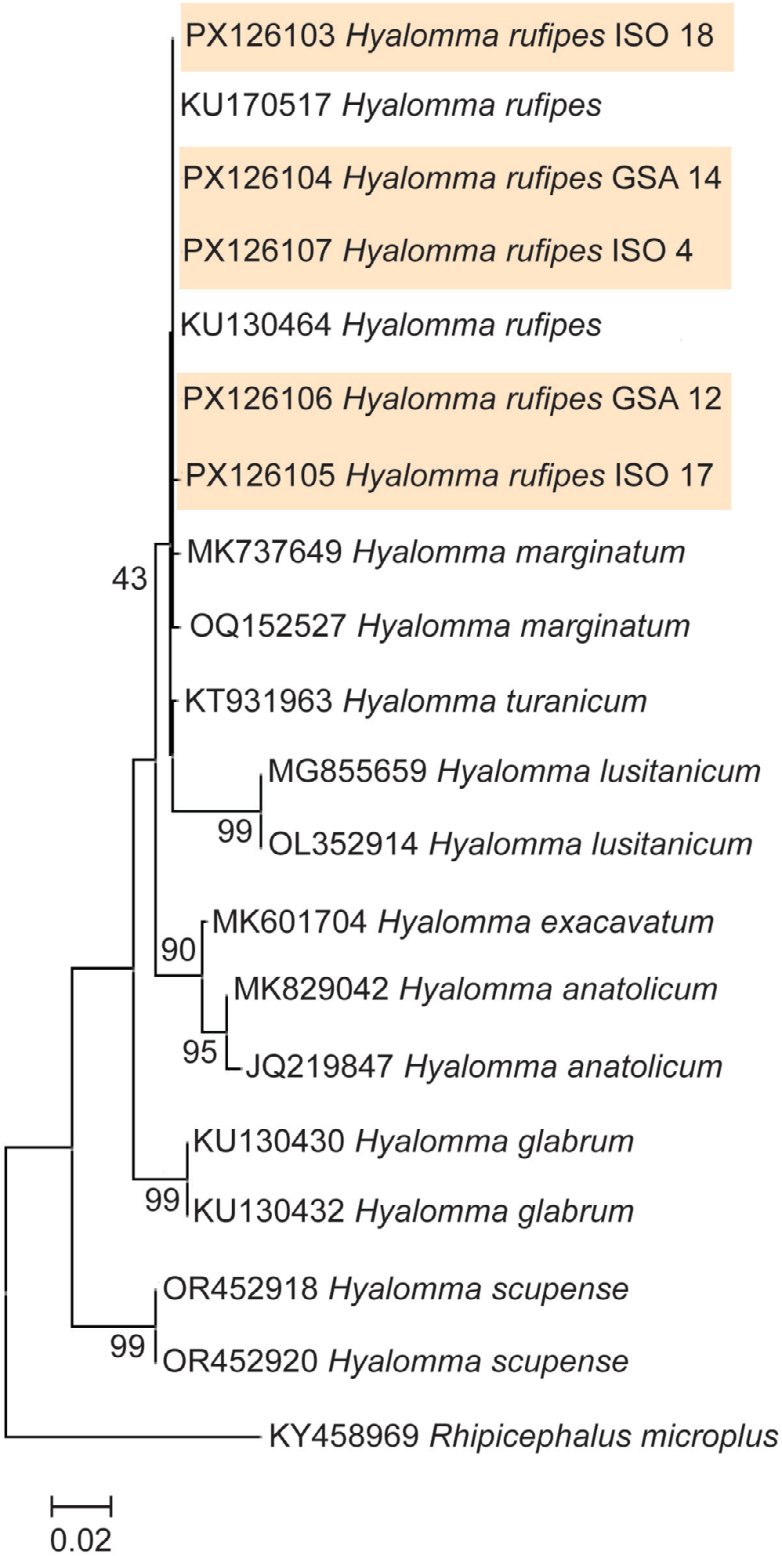
Maximum‐likelihood phylogenetic tree based on a segment of the 16S gene for the *H. rufipes* identified in this study. Bootstrap values are shown next to nodes on the tree. The scale bar at the bottom of the figure shows the evolutionary distance. The *H. rufipes* 16 s sequences from this study are indicated by the orange shading. The tick *Rhipicephalus microplus* was used as an outgroup for the tree. Genebank accession numbers are listed before the genus and species names in the tree.

*H. rufipes* morphological identification was confirmed by sequencing a partial region of the 16 s ribosomal gene. Of the five samples collected as part of this study, when sequenced, all form a distinct clade of *H. rufipes* with previously sequenced samples on Genbank (KU170517 and KU130464). However, two *H. marginatum* samples sequenced in previous studies also grouped in the same clade as all of the *H. rufipes* samples. All other *Hyalomma* species examined grouped in their own respective and distinct clades (Figure 1). All 16 s *H. rufipes* sequences from this study were deposited on Genbank (accession numbers PX126103‐PX126107).

### 3.2 Microsatellite Characterization

By using low pass sequencing of the *H. rufipes* genome with an Illumina platform, we obtained 5,608,135 reads with a mean read length of 150 bp, corresponding to a 1682 Mb genomic sequence of the *H. rufipes* . The raw sequence data are deposited in Genbank (project accession no. PRJNA1306167). Of the total number of reads, 162,635 contained one or more microsatellite loci (3%). Di‐nucleotide repeat motifs were the most abundant microsatellites in *H. rufipes* making up 60% of all microsatellites (File S1). Trinucleotide repeats were 18% and tetranucleotide repeats were 20% of all microsatellites (File S1). Among the 162,635 reads containing a microsatellites primer pairs were designed for 59,201 microsatellite loci. Of these, 30 loci were selected for PCR amplification and to determine rates of polymorphism (Table S1). Fourteen of the aforementioned 30 loci were amplified successfully and showed more than two alleles using samples from both the Garissa and Isiolo populations. Twelve out of fourteen loci identified were tetranucleotide repeats with the remaining two loci being a dinucleotide and a trinucleotide repeat (Table 1).

### 3.3 Population Genetic Analysis

All fourteen loci in both populations tested were observed to be polymorphic. Genotypes for each locus and individual tick are provided in File S2. The average number of alleles for all loci was 15.8 (Table 2). Locus 14 had the lowest allelic richness for both populations at 7.5 for Garissa and 6.1 for Isiolo (Table 2). All other loci had allelic richness higher than ten in both populations (Table 2). The loci tested had observed and expected heterozygosities ranging from 12 to 17 and 13.1 to 17.2, respectively (Table 2). Gene diversities were at 0.9 or above for twelve loci in both populations (Table 2). Table 2 shows the values for the global Hardy‐Weinburg exact tests with excess heterozygosity. Locus 14 is the only locus with a *p*‐value less than 0.05 in the Isiolo population (Table 2). The F_IS_ values for all loci in both populations were between −0.008 and 0.216 (Table 2), indicating low to moderate levels of inbreeding. Values near zero suggest that most loci are in Hardy–Weinberg equilibrium, while the slightly positive values at some loci point to a mild heterozygote deficiency, which may result from non‐random mating, population substructure, or the presence of null alleles. Null alleles were only detected in five loci. The five loci affected by null alleles were Hrms‐5, Hrms‐6, Hrms‐11, Hrms‐16, and Hrms‐21. The probabilities of null alleles in those loci are 0.1174, 0.1285, 0.0864, 0.089, and 0.0941, respectively. Stuttering and dropout were not detected in any loci.

**TABLE 2:** Characterization of 14 microsatellite loci for *H. rufipes* from Garissa and Isiolo populations in Kenya.

	N	N_all_	H_e_	H_o_	F_IS_	A_rich_	G_D_	*F*_ST_ Garissa vs Isiolo	HW
*Garissa*									
Hrms‐4	18	16	16.9	16	0.052	14.4	0.94	0.005	0.85
Hrms‐5	18	15	15.8	14	0.115	13.7	0.93	−0.008	0.97
Hrms‐6	18	14	16.6	12	0.283	12.7	0.93	0.089	0.99
Hrms‐7	18	15	16.9	17	−0.007	13.7	0.94	0.004	0.44
Hrms‐8	18	15	15.7	14	0.111	13.9	0.93	0.016	0.98
Hrms‐9	18	17	16.2	15	0.075	15.6	0.95	−0.001	0.95
Hrms‐10	18	16	16	14	0.128	14.7	0.95	0.004	0.89
Hrms‐11	16	17	14.9	13	0.131	15.7	0.94	0.006	0.97
Hrms‐13	18	21	17.3	17	0.017	18.1	0.96	−0.006	0.68
Hrms‐14	18	8	13.9	14	−0.008	7.5	0.77	0.005	0.45
Hrms‐16	18	13	14.6	14	0.045	12.2	0.92	0.001	0.64
Hrms‐21	18	15	16.5	13	0.216	13.3	0.92	0.011	1.0
Hrms‐24	17	15	15.1	15	0.009	14.3	0.95	0.012	0.86
Hrms‐28	18	12	15.8	16	−0.016	11.4	0.93	−0.006	0.49
Total	17.79	14.93	15.9	14.57	0.08	13.7	0.93	0.0093	0.80
*Isiolo*									
Hrms‐4	17	20	16.4	15	0.086	18.0	0.97	—	0.90
Hrms‐5	17	17	16.1	12	0.262	15.5	0.96	—	1.0
Hrms‐6	17	13	12.2	13	−0.067	11.4	0.72	—	0.28
Hrms‐7	17	16	15.5	14	0.102	14.6	0.92	—	0.97
Hrms‐8	18	18	17.1	15	0.128	16.0	0.96	—	0.98
Hrms‐9	18	21	16.5	15	0.094	18.9	0.97	—	1.0
Hrms‐10	18	20	16.2	16	0.01	17.6	0.95	—	0.85
Hrms‐11	17	18	16.2	13	0.203	16.3	0.96	—	1.0
Hrms‐13	18	19	17.1	17	0.007	16.4	0.95	—	0.76
Hrms‐14	18	7	12.0	17	−0.431	6.1	0.66	—	0.0004
Hrms‐16	18	16	15.2	12	0.214	15.2	0.95	—	1.0
Hrms‐21	18	18	16.6	16	0.035	15.2	0.92	—	0.73
Hrms‐24	16	15	13.2	12	0.093	15.0	0.95	—	0.64
Hrms‐28	18	16	16.7	16	0.046	14.6	0.93	—	0.89
Total	17.5	16.7	15.5	14.5	0.0559	15.0	0.91	—	0.79

*Note:* N_all_ is the number of alleles, H_e_ is the expected heterozygosity, H_o_ is the observed heterozygosity, F_IS_ is the inbreeding coefficient, A_rich_ is the allelic richness which measures number of alleles independent of sample size and G_D_ is gene diversity. HW are *p*‐values from the Hardy‐Weinburg exact tests for excess heterozygosity.

Pairwise *F*_ST_ values between the Garissa and Isiolo populations were uniformly low across all loci (*F*_ST_ < 0.1), indicating minimal genetic differentiation between the two groups. This low level of population structure suggests high gene flow and genetic similarity between the Garissa and Isiolo populations. The overall pairwise *F*_ST_ value for both populations encompassing all loci was 0.0093, suggesting little population differentiation (Table 2).

## 4 Discussion

Here we discuss the development of microsatellite markers for *H. rufipes*, which may provide a tool for future population genetics research focused on tracking tick movement and gene flow within the Kenyan‐Ugandan cattle corridor, with the goal of informing strategies to prevent Crimean‐Congo Hemorrhagic Fever Virus (CCHFV) outbreaks. To the best of our knowledge, no genetic markers have previously been characterized or developed for *H. rufipes* .

The 16 s sequencing results confirmed the morphological identifications for *H. rufipes* , and the phylogenetic tree results were consistent with expectations. Specifically, *H. rufipes* is part of a species complex that includes *H. marginatum* and *H. turanicum* (Uiterwijk et al. 2021; Estrada‐Peña et al. 2017; Rees et al. 2003). Low‐pass Illumina sequencing yielded a large and high‐quality genomic dataset for *H. rufipes* , from which a substantial number of microsatellite loci were identified (> 10,000). Most loci exhibited high allelic richness (> 10) and high gene diversity values (≥ 0.9 for twelve loci), suggesting that these markers are informative and suitable for assessing population structure and diversity. Locus 14 stood out with the lowest allelic richness in both populations and was the only locus to deviate significantly from Hardy–Weinberg equilibrium in Isiolo, potentially indicating locus‐specific effects such as selection, genotyping error, or population‐specific dynamics. Although a large set of primer pairs were designed, the final panel of 14 polymorphic loci demonstrated amplification success and high allelic diversity, making them suitable for population genetic studies. The observation that 12 of these loci were tetranucleotide repeats may reflect their higher stability and lower stutter rates compared to dinucleotides, which is advantageous for genotyping accuracy.

Population genetic analyses using the microsatellite markers developed in this study suggest there is a significant level of genetic variation in both the Garissa and Isiolo populations, with a mean allelic richness > 10 for most loci and gene diversities approaching 0.9. The majority of loci conformed to Hardy–Weinberg expectations, with only locus 14 in Isiolo showing significant deviation, possibly due to sampling effects or a small sampled population. Low to moderate *F*_*IS*_ values suggest that inbreeding is minimal in both populations sampled, and the detection of null alleles at only a small subset of loci, without overlap between populations, indicates that genotyping artifacts are unlikely to bias the overall results. Null allele presence is common within Ixodidae (Huber et al. 2019; De Meeûs et al. 2004; Koffi et al. 2006; Noel et al. 2012; Van Houtte et al. 2013). No loci were discarded from the analysis despite some of them having null alleles as all the frequencies were less than 15% (Van Oosten et al. 2014). The only loci with null alleles present were five, six, eleven, sixteen, and twenty‐one. The null allele frequencies for these loci were approximately 12%, 13%, and 9% for locus 11, 16, and 21, respectively. Null alleles were only detected within either Garissa or Isiolo populations; no locus had them in both populations. Locus 14 was the only locus with a *p*‐value of less than 0.05 suggesting it is not in Hardy‐Weinburg equilibrium (Table 2).

The observed low *F*_*ST*_ values across loci, coupled with an overall *F*_ST_ of 0.0093, indicate minimal population differentiation between Garissa and Isiolo, which are separated by approximately 250 km. Overall, the low levels of observed population differentiation and structure suggest there is gene flow between these geographically distinct sites, which could be driven by livestock movement within the Kenyan Ugandan cattle corridor, a well‐documented route for both animal and vector dispersal (Chiuya et al. 2021; Kilpatrick and Randolph 2012; Mossel et al. 2017; Nyaruaba et al. 2019). Genetic connectivity associated with potential tick movement has important epidemiological implications, as this could facilitate the spread and transmission of potential tick‐borne pathogens such as Crimean‐Congo Hemorrhagic Fever Virus (CCHFV). However, the sampling and genotyping of additional populations would be needed to further validate the hypothesis that tick movement and population connectivity are common in the Kenyan Ugandan cattle corridor.

Overall, these results demonstrate that the developed microsatellite panel is an important tool for assessing *H. rufipes* population structure. The high genetic diversity and low differentiation observed suggest that control measures targeting tick populations and/or disease control in one location are unlikely to be effective without considering regional‐scale livestock movement and tick dispersal and migration. Future studies incorporating broader geographic sampling and temporal monitoring will be essential to better understand the connectivity patterns of *H. rufipes* and their implications for tick‐borne disease spread in East Africa.

## Author Contributions

**Hamza Ahmad:** formal analysis (equal), investigation (lead), visualization (equal), writing – original draft (lead), writing – review and editing (equal). **Winnifred Aool:** investigation (supporting). **Victor Anyango:** investigation (supporting). **Teddy M. Nakayaki:** investigation (supporting). **Francis Mulwa:** investigation (supporting). **Betty Chelangat:** investigation (supporting). **Julius J. Lutwama:** resources (supporting), supervision (supporting). **Jonathan K. Kayondo:** investigation (supporting). **Martin Lukindu:** investigation (supporting), resources (supporting), supervision (supporting), writing – review and editing (supporting). **James Mutisya:** investigation (supporting). **Joel Lutomiah:** resources (supporting), supervision (supporting). **Lisa E. Hensley:** funding acquisition (equal), project administration (supporting). **Lee W. Cohnstaedt:** project administration (supporting), writing – review and editing (supporting). **Maria G. Onyango:** conceptualization (supporting), formal analysis (supporting), funding acquisition (equal), writing – review and editing (supporting). **Corey L. Brelsfoard:** conceptualization (lead), formal analysis (equal), funding acquisition (equal), investigation (supporting), methodology (lead), resources (lead), supervision (lead), visualization (equal), writing – review and editing (equal).

## Funding

This work was supported by a United States Department of Agriculture Cooperative Agreement (Agreement number: 58‐3022‐2‐023).

## Ethics Statement

This study was performed with approval from the Texas Tech University Institutional Biosafety Committee (IBC‐2023‐1041). Tick sample collection was carried out with approvals from the Uganda Virus Research Institute (UVRI, Ref. no. GC/127/949), the Uganda National Council for Science and Technology (UNCST, Ref. no. A296ES), the Kenya Medical Research Institute (KEMRI) Scientific Ethics Review Unit (SERU, Ref. no. 4684), and the National Commission for Science and Innovation (NACOSTI, Ref. no. 875261).

## Consent

No human subjects were used in this research.

## Conflicts of Interest

The authors declare no conflicts of interest.

## Supporting information


**Appendix S1:** ece373064‐sup‐0001‐AppendixS1.xlsx.

**Appendix S2:** ece373064‐sup‐0002‐AppendixS2.xlsx.

**Appendix S3:** ece373064‐sup‐0003‐AppendixS3.pdf.

## Data Availability

The that supports the findings of this study are available in the Supporting Information of this article and are available on Genbank [accession numbers KU170517 and KU130464; PX126103‐PX126107].
