# Supplementary material for: Development and Characterization of Microsatellite Genetic Markers for Hyalomma rufipes, a Tick Vector of Crimean‐Congo Hemorrhagic Fever Virus
Source: Ecol Evol. 2026 Feb 5;16(2):e73064. doi: 10.1002/ece3.73064 (PMC12875679; doi:10.1002/ece3.73064)
Supplement: Supplementary file 3 — Appendix S3: ece373064‐sup‐0003‐AppendixS3.pdf. [file ECE3-16-e73064-s003.pdf]

Loci name	Forward Primer 5'-3')	Reverse Primer (5'-3')	Repeat Motif and Count	Estimated Product Size (bp)	Annealing Temperature, °C
Hrms-1	GATGGCTGTGGCTACCTGTT	GCGTACGTGCTCGAATGTTC	$(AATC)_{12}$	175	55 °C
Hrms-2	CACCGAATTGCGCGTCTAAG	TCTTCCTTCCGACAAAGGCC	$(AAAC)_{10}$	184	55 °C
Hrms-3	GTAGAGGCCGTACGATGGTG	TCCATATGCACGTACCGCTC	$(AATG)_{15}$	202	55 °C
Hrms-4	ACATCAGCAACACAACGCAC	GGAGCCTACATAATGCGCCT	$(AAAC)_{13}$	188	55 °C
Hrms-5	TTACGCTCACAGTGACACCC	ATCGCGTGGCTACCTATGTG	$(AGAT)_{21}$	217	55 °C
Hrms-6	AAGCGATGGCAGTGTCGTTA	AACGTGACGCAGCAAGTTTC	$(AGAT)_{10}$	219	55 °C
Hrms-7	GGTCGTGTCAGCCAACCATA	GCCGTCAAACAAGGTGTCAC	$(AGAT)_{13}$	156	55 °C
Hrms-8	CGCCAACATCAGCAACACAA	AGGAGCCTACATAATGCGCC	$(AAAC)_{13}$	194	55 °C
Hrms-9	CAGCCGAGTACGATGTCCTC	TGACACCAGTGGCGGTATTC	$(AGAT)_{11}$	193	55 °C
Hrms-10	AACGGTGAGATGCATGGGTT	GCAAGTTTCAACGAACGCCT	$(AGAT)_{9}$	166	55 °C
Hrms-11	ATAGCGCACAGTACTCGAGC	AAAGCGGTGGATGCCTGTTA	$(AAT)_{13}$	153	55 °C
Hrms-12	AATGATGTTCTGGGACGCGT	TCCATATGCACGTACCGCTC	$(AATG)_{15}$	159	55 °C
Hrms-13	TCTAGCAGGGCTCAGGCTAA	ACCATTCGACCCTGCTTGAG	$(AAAG)_{8}$	144	55 °C
Hrms-14	ATGGCTGTAGCGATGGTACG	ACAACAGCTCCATTCTCCGG	$(AG)_{9}$	116	55 °C
Hrms-15	AATTAGGAAGGCTGCGCAGT	TCGAGGATGCCCAGAGAAGA	$(AG)_{8}$	141	55 °C
Hrms-16	GCGCCCTTCTCCTAACCTTT	CGAACCCACCTTCTTCGACA	$(AAAT)_{13}$	201	55 °C
Hrms-17	AATGATGTTCTGGGACGCGT	CTCCATATGCACGTACCGCT	$(AATG)_{15}$	160	55 °C
Hrms-18	AAGCAATCATTCCCGCCTCA	GAGACCAGCCTGAGCAACAT	$(AAAT)_{12}$	158	55 °C
Hrms-19	TACGGAGAGTCGGGACCATT	TGGTTCACTGTAGCCGCATT	$(AAT)_{9}$	177	55 °C
Hrms-20	CGACTTTCTGTGCTGGAGGT	ACTGATGGGAACGGAAGCTG	$(AAAT)_{10}$	143	55 °C
Hrms-21	AAGCGGAGTTCCCTAACACG	TAAGCTCGAACACGCTGGTT	$(AAAG)_{8}$	132	55 °C
Hrms-22	TGCCGTTTACTATGCCTGCA	CAACTCACACGTCCATGGGA	$(ATC)_{20}$	163	55 °C
Hrms-23	GCTGGGCCTTAAACTTTGGC	GCGCATTCTTCCTCGGGATA	$(ACAG)_{9}$	120	55 °C
Hrms-24	AAGCGGAGTTCCCTAACACG	GCAGTGGTATATCGCTGGCT	$(AAAG)_{8}$	167	55 °C
Hrms-25	TGCTTGAGCTGATGGAGGTG	CAATCGACTGGGAAGGCAGT	$(ACAT)_{17}$	202	55 °C
Hrms-26	TCTACCGCAAACCACCATCC	TAGCAGCTTCCTCAACCAGC	$(AAAG)_{13}$	219	55 °C
Hrms-27	TCATTTCAGTGCAGCGGTCT	AGCTCTCAATGCTTCGCTGT	$(AGAT)_{12}$	143	55 °C
Hrms-28	GGTCGTGTCAGCCAACCATA	GGGTTAACCGGTGTGGCATA	$(AGAT)_{13}$	198	55 °C
Hrms-29	GTGGAGATGTTGCGGAGTGA	AGCCTTGTGGTGTATCTGGC	$(AATG)_{13}$	187	55 °C
Hrms-30	AGACTGTTCCAGCTTCCTGC	AAGCTTAAGCACGGCGTACT	$(AAAG)_{10}$	207	55 °C
